# A library mobile device deployment to enhance the medical student experience in a rural longitudinal integrated clerkship

**DOI:** 10.5195/jmla.2019.442

**Published:** 2019-01-01

**Authors:** Emily M. Johnson, Carmen Howard

**Affiliations:** Assistant Professor and Regional Health Sciences Librarian, Library of the Health Sciences–Peoria, UIC Library, University of Illinois at Chicago, Peoria, IL, emj11@uic.edu; Instructor and Regional Health Sciences Librarian, Library of the Health Sciences–Peoria, UIC Library, University of Illinois at Chicago, Peoria, IL, choward4@uic.edu

## Abstract

**Objective:**

Investigators implemented the Rural Information Connection (RIC) project, a library-initiated deployment of iPad Mini 3s for third-year medical students who were enrolled in a seven-month rural longitudinal integrated clerkship (LIC) rotation. The research aims were to determine if devices preloaded with high-quality mobile health apps enhanced the experience and increased access to and awareness of mobile health information resources for the enrolled project participants.

**Methods:**

Nine participants enrolled in this mixed methods research project. Pre- and post-survey and structured learning journals (SLJs) were used for data collection on device and app use. Descriptive statistics and thematic coding analysis included data from seven pre-surveys, nine post-surveys, and sixty-four SLJ prompts. The validated Technology Acceptance Model instrument was also incorporated to gauge the devices’ integration into the participants’ workflow.

**Results:**

The investigation indicated that the iPad Mini 3 and resources were utilized and integrated at varying levels in the participants’ workflow. Reported use of health information apps suggests a preference for broad-based information sources rather than specific or specialized information resources. Participants performed several tasks on the device, including seeking background information, educating patients, and managing rotation schedules. Participant reflections indicated positive experiences utilizing the device and health information resources, which enhanced their rural LIC rotations.

**Conclusions:**

The research analysis demonstrates the information-seeking behavior of medical students immersed in a rural environment and indicates an acceptance of mobile technology into the workflow of participants in this project. Mobile device deployments offer great opportunities for librarians to design innovative programming in medical education.

## INTRODUCTION

Health care professionals practicing in rural environments often express the need for point-of-care health information, while simultaneously having limited access to the funding for resources that are required to answer their clinical questions [1–4]. Preparing medical students and residents prior to practicing in such an environment is critical to providing health care in rural communities [5]. Providing access to clinical information tools to achieve both educational and clinical care objectives presents an opportunity to support students who are engaged in training in longitudinal integrated clerkships (LICs) and rural environments using mobile technologies and relevant health information applications (apps) [6, 7].

In a 2016 news release, the Federal Communications Commission (FCC) stated that 39% of the rural population versus only 4% of the urban population lacked access to 25/3 megabits per second (Mbps) service, indicating that there continued to be a “persistent urban/rural disparity” in broadband Internet access [8]. This disparity also impacts physicians, small rural practices, and rural hospitals. According to the Assessment of Broadband Needs of Health Care Providers, rural providers were interested in using health information technologies to implement telehealth/telemedicine but lacked access to or had insufficient budgets to pay for the necessary broadband connections [8].

Broadband Internet access is not the only disparity associated with rural life. The National Rural Health Association reports that the primary care physician-to-patient ratio is significantly lower in rural areas than in urban areas [3]. Rural health professionals recognize that “professional isolation” is a factor contributing to reluctance to practice in rural settings [4]. One hypothesis is that information technology might aid in lessening the isolation that many in the rural health workforce feel [4].

All physicians have information needs that are unrelated to their locations, such as addressing their own knowledge gaps when faced with challenging medical cases, difficult treatment decisions, or unexpected results [9]. In their review of the literature, Clarke et al. found that primary care nurses and physicians shared patient-specific information needs regarding diagnosis, medication, treatment, epidemiology, prognosis, and etiology. Additionally, they found that for physicians and residents, diagnosis and treatment were the most common information needs [10]. Because community-based physicians practicing in rural areas frequently have multiple practice settings [11], mobile resources are well suited to fill these information needs for rural physicians and potentially lessen their sense of isolation.

The use of mobile devices in health care and health professions education has been well documented. In a 2017 survey by Healthcare Information and Management Systems Society Analytics, adoption rates of tablet devices to provide and coordinate patient care reached 79.8% among practicing physicians [12]. Several medical school curricula have taken advantage of this technology by incorporating mobile devices to enhance the educational experience, from preparing for examinations to information-seeking for patient care in traditional clerkships and LICs [6, 7, 13–20].

To support their users, many health sciences libraries are engaging with the mobile device environment by creating mobile app guides and websites, providing mobile device instruction, and lending devices for curriculum and personal use [21–27]. The authors, both faculty librarians, desired to support LICs and rural health care education, and based on our previous experience managing mobile devices and supporting mobile access to resources in an academic health sciences library, we developed a project called the “Rural Information Connection (RIC)” to improve access to high-quality mobile information resources for one of the most unique clerkships on campus.

The Consortium of Longitudinal Integrated Clerkships states that LICs require medical students to work with a clinician, providing comprehensive care to patients and meeting the majority of the core disciplinary clinical competencies in a prolonged experience [28]. The Rural Student Physician Program (RSPP), started at the University of Illinois College of Medicine at Peoria in 1996, is a seven-month LIC rotation during the third year of medical school that places students at a rural health care site with a practicing physician/preceptor. The RSPP program currently integrates a community-based research project and the disciplines of family medicine, internal medicine, pediatrics, obstetrics/gynecology, psychiatry, and general surgery.

RSPP students were selected to participate in the RIC project primarily because of the previously discussed challenges facing physicians in a rural environment and, specifically, due to the lack of resources that are available to rural physicians reported by the FCC and others [1–4, 8]. The RSPP was an optimal pilot partner for this device deployment due to the size of the student group and the lengthier time frame of the LIC. The smaller cohorts of four to five participants per year, plus the twenty-eight-week span, allowed more manageable mitigation of technology and policy issues. Additional factors included the flexibility and enthusiasm of the program director, faculty, and staff supporting the RSPP.

For the RIC project, iPad Mini 3s (hereafter referred to as iPad Minis) were obtained by the University of Illinois at Chicago’s Library of the Health Sciences–Peoria and preloaded with mobile apps to support the educational and clinical goals of the LIC [29]. We obtained the devices and some resources using grant funds from the National Network of Libraries of Medicine Greater Midwest Region. The grant pilot project showed positive indicators from participants, and we were asked to continue beyond the one-year grant-funded timeframe. This research study’s objectives were (1) to determine whether supplying preloaded iPad Minis with high-quality mobile health apps enhanced the experience of medical students who were enrolled in a rural student physician LIC and (2) to increase awareness of and access to mobile health information resources for clinical care in a rural environment.

## METHODS

### Design and participants

The target study population was third-year medical students enrolled in a rural LIC at a regional campus of an Illinois medical school. The iPad Mini lending project was deployed over two years from July 2015 to June 2017. Participants were enrolled in four separate cohorts for the seven-month LICs. We analyzed the effectiveness of the project using mixed methodology comprising paired pre- and post-surveys (supplemental Appendix A: Survey instrument) and thematic coding of structured learning journals (SLJs). This study was deemed exempt from review by the Institutional Review Board (IRB) at the University of Illinois College of Medicine at Peoria.

We designed the RIC project and secured funding, managed the devices, selected the resources and apps, and created a LibGuide to assist participants. One week prior to the rotation, participants attended an hour-long orientation in which we introduced the deployment and explained the research goals of the project. Participants received information about the resources and created required accounts for library-subscribed resources. Participants were asked to complete a paper consent document to confirm their willingness to participate and an iPad Mini loan document informing them that they would not be liable for any damage to or loss of the iPad Mini. No participants opted out of the RIC project. We administered hard-copy pre-surveys to gather demographic information, prior technology knowledge of the iPad Mini and resources, and anticipated technology use. As part of the survey, participants were provided a list of information resources and asked to identify resources that they had previously used, regardless of technology platform. Participants were given an opportunity to include additional resources that were not listed. The last section of the orientation focused on setting up personal accounts to access both clinical information resources and productivity tools.

To gather perceptions on technology use while in the rural LIC setting, participants were asked to complete eight SLJs. SLJs have been used as a “vehicle for reflection” in education [30]. RIC participants recorded brief experiences and reflections on their use of the iPad Minis and documented resources that they used during the LIC. By using this methodology, we anticipated gaining further insights into the use of iPad Minis in medical education and clinical practice in the rural environment. We used the Qualtrics survey tool [31] to send a prompt to the participant’s email every three to four weeks (supplemental Appendix B: Structured learning journal example). Participants were given a week to complete their responses. Participants were asked to spend no longer than twenty minutes on the prompts and to not include any identifiable patient information when answering the questions.

At the end of the 7-month LIC, participants attended a short meeting to return the iPad Minis and to complete a post-survey. The hard-copy post-survey included questions on iPad Mini use, questions on resource use during the rural LIC, and the Technology Acceptance Model (TAM) rating scale [32, 33]. In the post-survey, participants were asked to utilize a 4-point “frequency of use” scale reporting their adoption of each resource in the provided list. Participants were also asked to include additional resources that were not listed. The iPad Mini and resource questions from the post-survey were paired with the pre-survey to gauge the participants’ use during the LIC.

The TAM instrument has been used in previous research to evaluate mobile technology integration or deployments in educational and health care settings [32–40]. TAM measures the constructs of perceived usefulness (PU), perceived ease of use (PEU), and user acceptance (UA) of the technology in the participant’s workflow by attempting to explain the user’s “behavioral intention” with the iPad Mini technology, based on the theory of reasoned action [41]. The validated TAM instrument was modified for use in this study by inserting the device name, “iPad,” into the statement constructs to ascertain the adoption of this technology into the participants’ workflow.

### Data analysis

A total of 9 participants enrolled in the RIC project. Descriptive statistics were used to assess their responses from the closed-ended survey questions. The TAM instrument statements were presented on a Likert scale of 7 (extremely likely) to 1 (extremely unlikely). The TAM instrument was also assessed for its reliability utilizing Cronbach’s alpha with IBM SPSS Statistics version 22 [42].

Thematic coding was independently carried out by two investigators to analyze the responses in the SLJs. The first-level coding scheme identified resources used, tasks performed, and participant reflections on use of the device and the RIC project. A second-level of thematic coding grouped tasks into categories of information need, including both clinical and nonclinical student activities. Second-level coding of reflections focused on the benefits or challenges of broad topical areas (supplemental Appendix C: Thematic coding elements). The two investigators met following each level of coding to compare results and resolve differences via discussion until consensus was achieved.

## RESULTS

Analysis included data collected from participants who completed a rural LIC from July 2015 to June 2017 and incorporates seven pre-surveys, nine post-surveys, and sixty-four completed SLJs. A brief delay in obtaining IRB exemption prevented use of two early pre-surveys from being used in the analysis. Each participant completed an average of seven of the eight administered SLJ prompts. De-identified raw data from this study are available upon request.

### Demographics

Of the 9 total participants, 78% (n=7) were male, and 22% (n=2) were female. Participants identified their ethnicity as white (n=6, 67%), black or African American (n=1, 11%), or multi-race (n=2, 22%).

### Reported technology use

All participants reported owning a laptop or desktop computer and a smartphone with either an iOS or Android operating system at study enrollment. Five participants indicated using their laptops as their primary device for information gathering purposes in the pre-survey. Five participants also reported using a tablet, identifying the Apple iPad, Microsoft Surface, and Amazon Kindle as devices used.

In the post-survey, participants reported daily (n=6, 67%), weekly (n=1, 11%), or monthly (n=2, 22%) use of the iPad Mini devices during the 7-month LIC. Most participants (n=6, 67%) reported continuing use of a laptop as their primary device for gathering information in the post-survey.

The post-survey scores recorded for the TAM constructs were on a 7-point Likert rating scale (Table 1). Cronbach’s alpha was calculated between the 3 constructs of PU, PEU, and UA at α=0.958, suggesting some statements were redundant in the survey tool. When calculating the constructs individually, Cronbach’s alpha for PU and UA were both >0.98, which is too high to be determined reliable. Cronbach’s alpha for PEU was 0.893, making it the only reliable construct of the TAM tool for this investigation.

**Table 1:** Technology acceptance model post-survey scores

Technology Acceptance Model (TAM) constructs and statements	Average (mean)	Standard deviation
Perceived use (PU)		
Using the iPad in my work helps me to accomplish tasks more quickly	5.33	2.236
Using the iPad improves my work performance	4.89	2.027
Using the iPad increases my work productivity	4.78	2.048
Using the iPad enhances my effectiveness at work	5.22	2.224
Using the iPad makes it easier to do my work	5.11	2.147
I find the iPad useful in my work	5.33	2.236
Perceived ease of use (PEU)		
Learning to operate the iPad has been easy for me	6.78	0.441
I find it easy to get the iPad to do what I want it to do	6.56	0.726
My interaction with the iPad is clear and understandable	6.56	0.527
I find the iPad to be flexible to interact with	6.44	0.726
It is easy for me to become skillful at using the iPad	6.44	0.527
I find the iPad easy to use	6.67	0.500
User acceptance (UA)		
I use my iPad very frequently (many times per week)	5.67	2.693
I use my iPad for a variety of purposes (clinical notes, reports, medical info, etc.)	5.56	2.455

Most TAM responses reflected positively on the adoption of the iPad Mini technology into the participants’ workflow (averages >5 on the 7-point scale), apart from a slightly lower rating of the 2 PU measures on “work performance” and “work productivity” (averages <5). This might be due to the lack of access to electronic health records (EHRs) on the iPad Minis. The constructs of PU and UA showed wide variation in responses, whereas the construct of PEU showed narrow variation in this participant population.

### Reported resource use

Participants were asked to report their use of information resources in both their pre- and post-surveys and in their SLJs. Participants reported actual use of forty-four information resources, of which fifteen did not require continuous WiFi for use. Responses in each of the surveys and the SLJs were tabulated to determine the most frequently used resources (Table 2). The resource that participants identified as most used in all methodologies was UpToDate [43]. Other resources found in the top ten of all rankings were Medscape [44], YouTube [45], the Centers for Disease Control and Prevention (CDC) [46], and DynaMed Mobile [47]. Four additional resources were suggested in pre- and post-surveys to include on the iPad Minis. We added two suggested apps, MedCalc [48] and Omnio [49], and reviewed two others but did not include them because of lack of access and quality concerns. Participants frequently reported use of productivity tools, such as cloud storage or email software, as an information resource; these information tools were removed from the data analysis.

**Table 2:** Participant resource use

Rank	Pre-survey*	Structured learning journal†	Post-survey‡
1	UpToDate	UpToDate	UpToDate
2	Medscape§	DynaMed Mobile§	YouTube
3	YouTube	Uworld - USMLE	DynaMed Mobile§
4	Epocrates§	Wikipedia	Epocrates§
5	Centers for Disease Control and Prevention (CDC)	iBooks§	CDC
6	AccessMedicine	Drugs.com§	Medscape§
7	MedlinePlus	CDC	AccessMedicine
8	PubMed Mobile	Medscape§	ASCVD Risk Estimator Plus§
9	DynaMed Mobile§	GoodRx	DailyMed
10	UWorld - USMLE	YouTube	Drug Information Portal

* Ranking based on the number of participants that reported previously using this resource.

† Ranking based on the number of times that participants reported using this resource during their clerkship.

‡ Ranking based on a 4-point “frequency of use” scale.

§ Does not require continuous WiFi for use.

### Reported tasks

Tasks completed using the iPad Minis were reported in the pre- and post-surveys and in the SLJs. The pre-/post-survey contained pre-populated choices for selection, whereas the SLJ prompts were open responses. Participants’ use of the iPad Minis primarily fell into 2 domains: “clinical information needs” and “nonclinical student activities.” Participants’ survey responses regarding their anticipated and reported use of the iPad Minis are summarized in Table 3. Participants revealed high anticipated and reported use of the following clinical information needs: “read clinical information resources,” “answer clinical questions,” “show information to attendings,” and “show information to patients.” The participants disclosed anticipated and reported use of 2 nonclinical student activities: “perform other assignments” and “non-rotation related activities.” Participants anticipated using EHRs (n=3, 43%) and reviewing labs (n=5, 71%) but were unable to accomplish those tasks (n=0 for both tasks) due to some technical issues.

**Table 3:** iPad Mini reported tasks (pre-/post-survey)

iPad Mini reported tasks	Pre-survey anticipated use (n=7)	Percentage of responses (pre-survey)	Post-survey reported use (n=9)	Percentage of responses (post-survey)
“Read clinical information resources”	7	100%	9	100%
“Answer clinical questions”	7	100%	6	67%
“Show info to attendings”	5	71%	6	67%
“Show info to patients”	5	71%	6	67%
“Non-rotation related activities”	3	43%	5	56%
“Perform other assignments”	6	86%	4	44%
“Take notes”	1	14%	1	11%
“Access EHR”	3	43%	0	—
“Review labs”	5	71%	0	—

**Figure 1:**
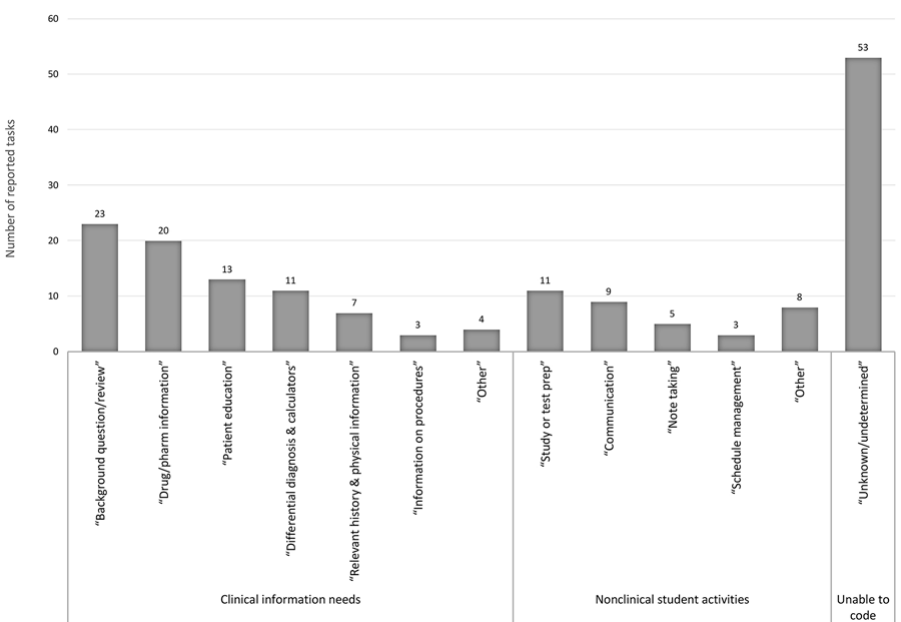
Participant iPad mini tasks (structured learning journal [SLJ] reported data)

Thematic coding of the SLJ responses identified 170 total tasks (Figure 1). Of these, 81 tasks were in the clinical information needs domain, and 36 tasks were in the nonclinical student activities domain. Due to the open-ended nature of the SLJs, there were 53 responses that were identifiable as a task but for which we were unable to determine the nature of the information need. The most frequently reported clinical information need was answering “background questions/review” of general information. This need was followed closely by the use of “drug and pharmacology information,” “patient education” materials, and “differential diagnosis and calculators.” Participants also reported using the iPad Minis to look up “information on procedures” and “relevant history and physical information.” Participants described using the iPad Minis for a variety of nonclinical student activities, including “studying or test prep,” “communication” with peers/preceptors/faculty, “note taking,” and “schedule management.” Additionally, participants reported using their devices for activities related to the research project that is a component of the LIC.

### Reported reflections on use

**Figure 2:**
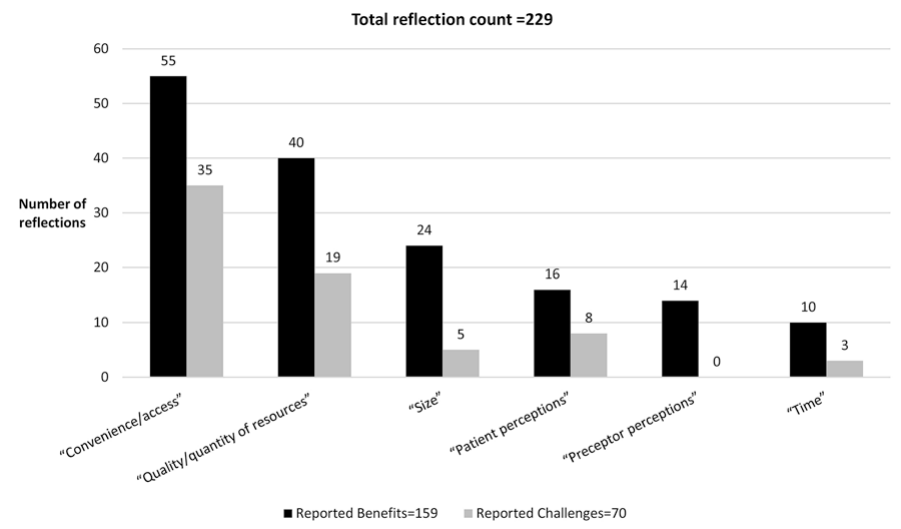
Participant iPad mini use reflections (SLJ reported data)

Participants were prompted in the SLJs to reflect upon aspects of their iPad Mini device use and upon the RIC project as a whole (Figure 2). Thematic coding identified 264 total reflections. Of these, 229 reflected specifically on iPad Mini use and 35 reflected on the RIC project more generally. Use reflections were grouped into 2 domains (“benefits” and “challenges”) and six categories (“preceptor perceptions,” “patient perceptions,” “time,” “size,” “convenience/access,” or “quality/quantity of resources”). We determined that 159 reflections expressed benefits and 70 expressed challenges. No participants reported that their preceptors had a negative perception of device use. However, there were some concerns that patients might see the device in a negative way, although benefits were still reported at twice the rate of challenges. Both “time” and “size” were reported as benefits of the device. Benefits were reported 1.5 times as often as challenges in the category of “convenience/access” and more than twice as often in the category of “quality/quantity of resources.” Specific reflections included “On a daily basis I use my [iPad Mini] to look up clinical information using DynaMed [Mobile], Google, and UpToDate” and “The iPad [Mini] definitely has great functionality in providing convenience for use in multiple settings.”

## DISCUSSION

Through analysis of the pre-/post-survey and SLJs, we showed that the RIC project enhanced the student experience in the rural LIC and increased the access to and awareness of mobile health resources in a rural environment. This enhancement is evident in the adoption of the iPad Mini into the majority of participants’ workflow, the reported activities performed on the devices, and the reported benefits of the devices outweighing the reported challenges. Participants had access to mobile health information resources throughout the entire seven-month rural LIC and reported utilizing familiar resources and integrating new resources into their clinical and nonclinical activities.

Throughout the project, the participants reflected on their primary uses and on the convenience of the iPad Minis; for example, “Primary uses were for researching differential diagnoses, confirming medication doses, generic vs brand name prescription availability, and studying medical literature.” These responses in our findings corroborated the use of mobile devices and tablets for information-seeking needs and management of scheduling logistics in undergraduate medical education (UGME) and rural clinical practice [1, 14, 50, 51]. Our findings also indicate that a high level of library integration into a mobile device deployment deserves the investment of library financial and staff resources and can lead to increased inclusion of librarians and information services in UGME and clinical practice. Specifically at our institution, this mobile device deployment not only fully integrated the library into the LIC, but also led to librarian involvement in a UGME taskforce in the College of Medicine.

The RIC participants reported 159 benefits and 70 challenges in the SLJ responses. With more than twice as many reported benefits as challenges, the RIC project can claim an overall positive value for the participants. A participant nicely summarized the value with this reflection: “Using a tablet has been very beneficial to me out in my community rotation. I am able to access research articles and protocols quicker than if I had to sit down with a [desktop] computer. I felt that using DynaMed [Mobile], UpToDate, and some of the pharmacy drug references contributed to my care of my patients in clinic and the hospital.” The overall positive value of the project is also reflected in the RSPP clerkship director’s request to continue the RIC beyond the pilot and to fully integrate the project into the RSPP experience.

The survey and SLJ data analysis give insights into the type of tasks undertaken with the iPad Minis. The survey presented broader task categories for the participants to select, whereas the open response of the SLJ allowed for more granularity in descriptions of tasks. We conceptually mapped the following categories from the survey to the SLJ coded tasks: survey response “read clinical information” mapped to coded tasks of “background question/review” and “relevant history and physical information”; survey response “answer clinical question” to coded tasks “drug/pharmacology information,” “procedure information,” and “differential diagnosis and calculators”; and survey response “show information to patients” to coded task “patient education.” The use of the iPad Minis for clinical information was expected and is well documented in previous literature [6, 16, 21, 52].

An unexpected result was a change in the use of the iPad Minis for patient education between cohorts during the two-year project. The first cohort of participants expressed that patients would possibly negatively perceive the participants’ professionalism, including the following sample SLJ response: “I will refrain from using my [iPad Mini] when interacting with a patient. I worry that it sends the message that I am not listening to them.” However, the next three cohorts expressed fewer negative perceptions and indicated positive uses, such as providing examples of using growth charts in a well child visit, showing disease diagrams, and calculating cardiovascular disease risk during the patient encounter. Based on the participant reflections, we now emphasize patient education use during the orientation and added patient education resources to the iPad Mini, such as the apps Essential Anatomy 5 [53] and Pill Identifier by Drugs.com [54]. Markman et al. also documented potential opportunities for medical students to use mobile resources for patient education [55].

We also noted that participants anticipated using EHRs on the iPad Minis to access information and review lab results for patient care at their rural placement. The Citrix Receiver app [56], which allows virtual access to desktops and EHRs on the iPad Minis, was preloaded onto the devices. However during the LIC, participants discussed problems with compatibility with their sites’ EHRs and Citrix Receiver’s interface, which had limited functionality for documentation. The educational objectives of clinical clerkships emphasized appropriate documentation of clinical encounters in the patient record [57], and the iPad Mini technology prohibited accomplishing this task. This access issue might be a reason why the participants indicated a lower rating on the TAM scale for “improves my work performance” and “increases my work productivity.” Unfortunately, we have limited contact with the rural placement sites, so participants must rely on other access points to the specific EHR for their documentation needs.

Participants’ responses to the surveys and SLJs also provided insight into resource use. One observation regarding resource use was that participants tended to use all-in-one or broad-based resources, like UpToDate and DynaMed Mobile, to fulfill their clinical information needs. It is possible that medical students, whose needs are more general, might not use specialized and highly specific resources as frequently as physicians and residents do. This hypothesis was supported by the coding analysis, which showed high representation of background information needs.

Increased knowledge and use of the DynaMed Mobile resource was of particular note. DynaMed Mobile ranked ninth among resources previously used in the pre-survey responses, second in the frequency of use as reported in SLJ responses, and third in the frequency of use reported during the post-survey. This reported increase in use might be attributable to the orientation and training at the beginning of the LIC. Another possible reason for the increase might be due to the ability to use DynaMed Mobile without an Internet connection as the content resides on the device as part of the app. Participants did report issues with WiFi and Internet connectivity as challenges (n=3) during their LICs, confirming a known challenge in the rural environment [1, 8].

Additionally, participants used the iPad Minis to email their peers, administrators, and faculty as reported in the SLJs; for example, “I also am able to readily answer emails from school or from student organizations that I am [a part] of at school.” By supplying rural student physicians with another access point to their colleagues, utilizing the mobile devices may effectively help with the sense of isolation that rural physicians commonly feel [4]. One participant reflected: “I also use the device for keeping up and looking up my monthly schedule with [my preceptor] and for addressing emails.” The mobile device has promise as that access point to support networks, providing students access to virtual communities of practice and utilizing social coping mechanisms to mitigate commonly felt issues in the rural setting [58]. This area of research is emerging, and librarians can be a key asset to establishing and participating in these virtual communities.

### Lessons learned

Several implications for librarians should be considered before implementing a similar mobile device deployment program. Our partnership with LIC education provided a unique audience due to the length of time of the rotation and the wide variety of information needed. The information needs of the participants in this group extended beyond the clinical content that might be needed during a typical rotation to include patient education, communication, and test preparation materials.

Many studies have advocated for creating specialty mobile library guides [21, 52], and we stress the importance of engaging the audience in creating these resource lists during the time of need. By engaging participants throughout the LIC, we captured feedback showing a range of clinical and nonclinical information needs.

The RIC project produced many technology challenges including time-consuming program implementation that required the skills of librarians and/or technology experts. The device management software selected for this deployment was Apple Configurator 2, which added to the challenges because it was dependent on the operating system [59]. Librarians who are considering implementing a similar program should look carefully at the systems in place in the library and how the systems will communicate with the mobile device systems, the availability of apps for devices with different operating systems, and what best meets the needs of students without becoming unmanageable for the librarians.

Purchasing resources can also be a challenge at some institutions due to the varied nature of app licensing. Many apps and/or electronic books and journals operate on a yearly license that may not be feasible for grant or institutional budgets.

Throughout the RIC project, we observed that some participants seemed more motivated to explore the devices and integrate them into their workflow. These participants were more apt to implement the iPad Minis in the clinical setting and use them for their various tasks. This motivation has been noted in several mobile device deployments in medical education [6, 7, 14, 15] and is reflected by the variation in responses for the TAM constructs of PU and UA among our participants. Those who did not integrate the iPad Minis into their workflow indicated use of other technology for their clinical information and nonclinical information needs, either a computer or smartphone. Librarians should acknowledge that it is important to “accept variability” among students in use of the technology [60].

Finally, libraries do not necessarily need to purchase mobile devices. However, this approach creates consistency amongst the devices and requires a narrower range of expertise for the librarians. Libraries that do not purchase devices would likely need to assist students in finding, downloading, and otherwise setting up their personal devices to make optimal use of clinical information resources, both the library’s resources and those outside of traditional collection development.

### Study limitations

There were limitations to this study. The small sample size represented a small institutional cohort, and there was no control group. Participants were asked to report their own behavior, and, as such, the behaviors reported might not represent actual practice or technology use. Cronbach’s alpha calculations for the TAM constructs of PU and UA were too high to be deemed reliable. Some free-form SLJ entries were vague and did not provide the granularity to categorize and, thus, were added to the “unknown/unable to categorize” code in the scheme because the true intentions or feelings behind those responses were unknowable. As previously mentioned, a slight delay in IRB exemption prevented early pre-survey data for two participants from being included in the analysis.

## CONCLUSION

The iPad Mini deployment and research analysis showed the information-seeking behavior of medical students immersed in a rural LIC environment and their acceptance of new technology into their workflow. Using the iPad Minis, the participants accessed essential clinical information, experienced improved patient education interactions, and accessed tools and resources to assist them in their rural LIC experiences. The use of a mobile device enhanced their rural LICs by providing needed information resources and a connection to the campus community. A positive effect was indicated by the research analysis and the RSPP clerkship director’s request to integrate the project into the rural LIC beyond the original pilot. RIC continues to be managed by library faculty.

Librarians’ participation in mobile device deployments extends support of the just-in-time information need and, in this case, gives a beneficial experience for medical students in the rural environment LIC. The continued ubiquity of mobile devices supplies great opportunities for librarians to join the culture of innovative mobile device programming in health care and health sciences education.

## SUPPLEMENTAL FILES

Appendix A Survey instrument

Appendix B Structured learning journal example

Appendix C Thematic coding elements
