# Supplementary material for: A library mobile device deployment to enhance the medical student experience in a rural longitudinal integrated clerkship
Source: J Med Libr Assoc. 2019 Jan 1;107(1):30–42. doi: 10.5195/jmla.2019.442 (PMC6300226; doi:10.5195/jmla.2019.442)
Supplement: Appendix A [file jmla-107-30-s001.pdf]

JMLA Journal of the Medical Library Association

SC

Supplemental content to
J Med Libr Assoc. Jan;107(1):dx.doi.org/10.5195/jmla.2019.442
www.jmla.mlanet.org
© Johnson, Howard 2019

A library mobile device deployment to enhance the medical student experience in a rural longitudinal integrated clerkship

Emily M. Johnson, AHIP; Carmen Howard

APPENDIX A

Survey instrument

Rural Information Connection: pre-rotation survey

Survey introduction:
This survey will take you approximately 15 minutes to complete. Information from this survey will be used to evaluate and improve the implementation of the Rural Information Connection pilot project. Although we request your name to facilitate pre/post comparisons, your identity and responses will be kept confidential.

Thank you for participating!

Name: ____

Date: ____

What is your gender?
A. Male
B. Female
C. Do not want to disclose

What is your ethnicity? [Please circle all that apply]
A. American Indian or Alaska Native
B. Asian
C. Black or African American
D. Hispanic or Latino/a
E. Native Hawaiian or Other Pacific Islander
F. White
G. Other

What technology do you currently own?

Technology	Yes	No	Don't know	Type/Brand? [Write in response]
A. Desktop computer?				
B. Laptop computer?				
C. Smartphone with Internet browsing ability?				
D. Tablet device (netbook, iPad, Kindle, etc.)				

SC
Supplemental content to
J Med Libr Assoc. Jan;107(1):dx.doi.org/10.5195/jmla.2019.442
www.jmla.mlanet.org
© Johnson, Howard 2019

How do you expect to use the iPad during the rotation? [Circle all that apply]

A. Read clinical information on the iPad
B. Take notes
C. Answer clinical questions
D. Log into the electronic health record (EHR)
E. Review labs on rounds
F. Complete other medical school activities
G. Show information to patients
H. Show information to attending physicians/residents
I. Non-course related activities
J. Other [Please specify] ____

Health sciences resource use

Which of the following databases/clinical tools have you previously used on any platform (i.e., computer, tablet, smartphone)?

Databases/clinical tools	Yes	No	I do not know what this resource is
AccessMedicine			
ACP Clinical Practice Guidelines			
ACP Immunization Advisor			
AFP by Topic			
AIDSinfo Mobile			
American Academy of Family Physicians			
ASCVD			
Browzine			
Calculate by QxMD			
Canopy Medical Translator			
CDC			
Citrix			
ClinicalKey			
Clinical Pharmacology			
DailyMed			
Drugs.com			
DynaMed			
Embryo			
Epocrates Basic			
ePSS			
Figure 1			
GoodRX			
Kindle			
LactMed			
LexiComp			
MDCalc			
Medical Letter			
MedlinePlus			
Medscape			
MicroMedex			
Omnio			
PHPartners			
Prognosis			

Databases/clinical tools	Yes	No	I do not know what this resource is
PubMedMobile			
Read by QxMD			
TouchSurgery			
TOXNET Mobile			
UpToDate			
UWorld USMLE			
WebMD for iPads			
YouTube			

What additional databases or clinical tools would you like to see on the iPad for your use?

____

Technology use questions

Definition: Information gathering: technology use for the purpose of finding needed information

In an average month in medical school, I use technology for information gathering purposes…
A. Daily
B. A couple days a week
C. Weekly
D. Monthly
E. Never
F. X. Unclear if this applies to me

What is the primary device you used for information gathering purposes?
A. Desktop computer
B. Laptop computer
C. Smartphone device
D. Tablet device
E. Other [Please specify] ________________________________

Overall, technology makes my life easier.
A. Strongly agree
B. Agree
C. Neither agree nor disagree
D. Disagree
E. Strongly disagree

I feel I accomplish more in my academic clinical life because of technology.
A. Strongly agree
B. Agree
C. Neither agree nor disagree
D. Disagree
E. Strongly disagree

SC
Supplemental content to
J Med Libr Assoc. Jan;107(1):dx.doi.org/10.5195/jmla.2019.442
www.jmla.mlanet.org
© Johnson, Howard 2019

If I don’t use technology for a week, I feel bothered or out of sorts.
A. Strongly agree
B. Agree
C. Neither agree nor disagree
D. Disagree
E. Strongly disagree

Given my use of all technologies at my disposal, on average I feel I am a(n):
A. Expert user
B. Advanced user
C. Intermediate user
D. Basic user
E. Non-technology user

JMLA Journal of the Medical Library Association

SC

Supplemental content to
J Med Libr Assoc. Jan;107(1):dx.doi.org/10.5195/jmla.2019.442
www.jmla.mlanet.org
© Johnson, Howard 2019

Rural Information Connection: post-rotation survey

Survey introduction:
This survey is a follow-up to the technology use survey you completed at the beginning of the rotation. It is intended to update information on your use of technology and the iPad over the rotation. Information from this survey will be used to evaluate and improve the implementation of the Rural Information Connection pilot project.

Although we request your name to facilitate pre/post comparisons, your identity and responses will be kept confidential.

This survey takes approximately 25 minutes to complete.

Thank you for participating!

Name: ____

Date: ____

Did you use the iPad during your rotation?
A. Yes
B. No

How often did you use the iPad?
A. Daily
B. A couple days a week
C. Weekly
D. Monthly
E. Never
F. X. Unclear if this applies to me

How did you use the iPad during the rotation? [Circle all that apply]
A. Read clinical information on the iPad
B. Took notes
C. Answered clinical questions
D. Logged into EHR
E. Reviewed labs on rounds
F. Completed other medical school assigned activities
G. Showed information to patients
H. Showed information to attending physicians/residents
I. Non-course related activities
J. Other [Please specify] ____

Journal of the Medical Library Association

Supplemental content to
J Med Libr Assoc. Jan;107(1):dx.doi.org/10.5195/jmla.2019.442
www.jmla.mlanet.org
© Johnson, Howard 2019

Technology Acceptance Model section

Technology Acceptance Model questionnaire

	**Likely**			**Neutral**			**Unlikely**
**Scale**	**Extremely**	**Quite**	**Slightly**	**Neither**	**Slightly**	**Quite**	**Extremely**
Using the iPad in my work helps me to accomplish tasks more quickly							
Using the iPad improves my work performance							
Using the iPad increases my work productivity							
Using the iPad enhances my effectiveness at work							
Using the iPad makes it easier to do my work							
I find the iPad useful in my work							
Learning to operate the iPad has been easy for me							
I find it easy to get the iPad to do what I want it to do							
My interaction with the iPad is clear and understandable							
I find the iPad to be flexible to interact with							
It is easy for me to become skillful at using the iPad							
I find the iPad easy to use							
I use my iPad very frequently (many times per week)							
I use my iPad for a variety of purposes (clinical notes, reports, medical info, etc.)							

JMLA Journal of the Medical Library Association

SC
Supplemental content to
J Med Libr Assoc. Jan;107(1):dx.doi.org/10.5195/jmla.2019.442
www.jmla.mlanet.org
© Johnson, Howard 2019

Health sciences resource use section

Which of the following databases/clinical tools have you used during your rotation on the iPad?

Databases/clinical tools	More than once per week	Once per week	Once during the term	Never
AccessMedicine				
ACP Clinical Practice Guidelines				
ACP Immunization Advisor				
AFP by Topic				
AIDSinfo Mobile				
American Academy of Family Physicians				
ASCVD				
Browzine				
Calculate by QxMD				
Canopy Medical Translator				
CDC				
Citrix				
ClinicalKey				
Clinical Pharmacology				
DailyMed				
Drugs.com				
DynaMed				
Embryo				
Epocrates Basic				
ePSS				
Figure 1				
GoodRX				
Kindle				
LactMed				
LexiComp				
MDCalc				
Medical Letter				
MedlinePlus				
Medscape				
MicroMedex				
Omnio				
PHPartners				
Prognosis				
PubMedMobile				
Read by QxMD				
TouchSurgery				
TOXNET Mobile				
UpToDate				
UWorld USMLE				
WebMD for iPads				
YouTube				

SC
Supplemental content to
J Med Libr Assoc. Jan;107(1):dx.doi.org/10.5195/jmla.2019.442
www.jmla.mlanet.org
© Johnson, Howard 2019

What additional databases or clinical tools would you have liked to see on the iPad for your use?

Please provide at least one example of which you feel your use of the iPad was effective in this rotation.

Please provide at least one example of which you feel your use of the iPad was ineffective or was limited in completing a task in your rotation.

Technology use questions

1. In an average month in medical school, I use technology for information gathering purposes…
A. Daily
B. Weekly
C. Less than weekly
D. Never
E. Unclear if this applies to me

2. What is the primary device you used for information gathering purposes?
A. Desktop computer
B. Laptop computer
C. Smartphone device
D. Tablet device
E. Other [Please specify] ______________________

3. Overall, technology makes my life easier.
A. Strongly agree
B. Agree
C. Neither agree nor disagree
D. Disagree
E. Strongly disagree

4. I feel I accomplish more in my academic clinical life because of technology.
A. Strongly agree
B. Agree
C. Neither agree nor disagree
D. Disagree
E. Strongly disagree

5. If I don’t use technology for a week, I feel bothered or out of sorts.
A. Strongly agree
B. Agree
C. Neither agree nor disagree
D. Disagree
E. Strongly disagree

JMLA Journal of the Medical Library Association

SC
Supplemental content to
J Med Libr Assoc. Jan;107(1):dx.doi.org/10.5195/jmla.2019.442
www.jmla.mlanet.org
© Johnson, Howard 2019

6. Given my use of all technologies at my disposal, on average I feel I am a(n):

F. Expert user
G. Advanced user
H. Intermediate user
I. Basic user
J. Non-technology user
