# Supplementary material for: A library mobile device deployment to enhance the medical student experience in a rural longitudinal integrated clerkship
Source: J Med Libr Assoc. 2019 Jan 1;107(1):30–42. doi: 10.5195/jmla.2019.442 (PMC6300226; doi:10.5195/jmla.2019.442)
Supplement: Appendix B [file jmla-107-30-s002.pdf]

JMLA Journal of the Medical Library Association

SC
Supplemental content to
J Med Libr Assoc. Jan;107(1):dx.doi.org/10.5195/jmla.2019.442
www.jmla.mlanet.org
© Johnson, Howard 2019

A library mobile device deployment to enhance the medical student experience in a rural longitudinal integrated clerkship

Emily M. Johnson, AHIP; Carmen Howard

APPENDIX B

Structured learning journal example

Rural Information Connection learning journal 1

[Start of Block: Default Question Block]

Q1. Rural Information Connection Project
Structured reflective learning journal prompt one

This structured reflective learning journal prompt will tell us about your experiences utilizing the iPad Mini during your rural rotation. Please reflect on your use of the iPad these past weeks to write your reflection.

Your responses will help us in evaluating this project and improving it in further iterations for your peers.

Definition: Reflective Learning Journal: Reflective journal is designed to help you think deeply about your learning, especially on issues such as: your progress in learning, the difficulties you encountered in the process of learning, the strategies you have taken to get around those difficulties, and your evaluation of your own performance.

Q6. Name:

____

Q12. What setting were you in during these last three weeks: (select all that apply)
O In-patient hospital (1)
O Out-patient clinic (2)
O Nursing home (3)
O Other: (4) ____

Q2. Did you use the iPad Mini in the past couple of weeks in your work on your rotation?
O Yes (1)
O No (2)

[Skip To: Q3 If Q2=Yes]
[Skip To: Q5 If Q2=No]

Q3. During the introductory weeks of your rural rotation, how did you use the iPad mini? Please describe one scenario that you feel the iPad mini enhanced your learning or clinical experience:

____

SC
Supplemental content to
J Med Libr Assoc. Jan;107(1):dx.doi.org/10.5195/jmla.2019.442
www.jmla.mlanet.org
© Johnson, Howard 2019

Q4. Please list some of the resources you used during the last three weeks:

[Skip To: Q10 If Q4 Is Not Empty]
[Skip To: Q10 If Q4 Is Empty]

Q5. During your introductory weeks of your M3 year and rural rotation, what barriers do you perceive prevented you from using the iPad mini? Examples of barriers: Time, Professionalism, Internet Connectivity, Resource Limitations, Lack of Training, etc.

[Skip To: Q10 If Q5 Is Empty]
[Skip To: Q10 If Q5 Is Not Empty]

Q10. To help you learn about the clinical applications of some of the apps loaded on your iPad, I would like to prepare some short descriptions about the apps. To target these to your needs, I will be asking for feedback on the format and delivery of this information. These would hopefully take no longer than 10 minutes of your time for review.

Q9. What would you like to learn about the apps? (Select all topics of interest)
O Featured app write up with screen shots of content (3)
O Short video capture of features of an app (1)
O Write up working through a primary care case with app resources (2)
O Key criteria to review to evaluate apps (4)
O Strategies to integrate the iPad into your workflow (5)
O Other: (6) ____

Q8. To learn more about the resources on the iPad, what would be your preferred method of receiving that information?
O Blog feed (RSS) (1)
O Email message specifically about the resource (2)
O Link included in email for structured learning prompts (3)

Q11. If you have any questions, comments, concerns about the iPad Rural Student Physician Program (RSPP) Project, please provide them in the follow box.

I will respond to your questions as soon as possible.

[End of Block: Default Question Block]
