# Supplementary material for: A library mobile device deployment to enhance the medical student experience in a rural longitudinal integrated clerkship
Source: J Med Libr Assoc. 2019 Jan 1;107(1):30–42. doi: 10.5195/jmla.2019.442 (PMC6300226; doi:10.5195/jmla.2019.442)
Supplement: Appendix C [file jmla-107-30-s003.pdf]

Journal of the Medical Library Association
Supplemental content to
J Med Libr Assoc. Jan;107(1):dx.doi.org/10.5195/jmla.2019.442
www.jmla.mlanet.org
© Johnson, Howard 2019

A library mobile device deployment to enhance the medical student experience in a rural longitudinal integrated clerkship

Emily M. Johnson, AHIP; Carmen Howard

APPENDIX C

Thematic coding elements

Tasks:

- Clinical information needs:
  - Drug/pharmacology information
  - Information on procedures
  - Differential diagnosis and calculators
  - Patient education
  - Background information/question and/or memory refresh
  - Relevant history and physical information
  - Other [clinical information need was clear but does not fall into a predefined category]
  - Unknown/Unable to determine information sought [information need is unclear]
- Nonclinical student activities/information needs:
  - Study or test preparation
  - Communication
  - Schedule management
  - Note taking
  - Other [Nonclinical activity/information need was clear but does not fall into a predefined category]
  - Unknown/Unable to determine activity [Activity/information need is unclear]

Reflections:

- iPad use: benefits
  - Professionalism
    - Preceptor perceptions of device use
    - Patient perceptions of device use
  - Time
  - Technology
    - Size
    - Convenience/access
    - Quality/quantity of resources
- iPad use: challenges
  - Professionalism
    - Preceptor perceptions of device use
    - Patient perceptions of device use
  - Time
  - Technology
    - Size
    - Convenience/access
    - Quality/quantity of resources

JMLA Journal of the Medical Library Association
SC
Supplemental content to
J Med Libr Assoc. Jan;107(1):dx.doi.org/10.5195/jmla.2019.442
www.jmla.mlanet.org
© Johnson, Howard 2019

- RIC iPad lending program
  - Training
  - Resource/technology duplication (already have access through phone, computer, etc.)

Resources:

- Activity reported:
  - In pre-survey (reported previous use of resources)
  - In structured learning journals (reported current activity)
  - In post-survey (reported activity in past seven months)
